# Patterns of Anti-Osteoporosis Medication Use among Women at High Risk of Fracture: Findings from the Global Longitudinal Study of Osteoporosis in Women (GLOW)

**DOI:** 10.1371/journal.pone.0082840

**Published:** 2013-12-20

**Authors:** Stephen Gehlbach, Frederick H. Hooven, Allison Wyman, Adolfo Diez-Perez, Jonathan D. Adachi, Xuemei Luo, Andrew G. Bushmakin, Frederick A. Anderson, for the GLOW Investigators

**Affiliations:** 1 University of Massachusetts Medical School, Worcester, Massachusetts, United States of America; 2 Hospital del Mar, Autonomous University of Barcelona, Spain; 3 Saint Joseph’s Hospital, McMaster University, Hamilton, Ontario, Canada; 4 Pfizer, Groton, Connecticut, United States of America; Georgia Regents University, United States of America

## Abstract

**Objective:**

To assess patterns of anti-osteoporosis medication (AOM) use over 3 years among women at high risk of major fracture.

**Methods:**

The GLOW registry follows a cohort of more than 40,000 women aged ≥55 from 615 primary care practices in 10 countries. Self-administered surveys (baseline, 12, 24, and 36 months) collected data on patient characteristics, perception of fracture risk, and AOM use. FRAX scores were calculated from the baseline surveys and women classified as high risk if their FRAX 10-year probability of major fracture was ≥20%.

**Results:**

A total of 5774 women were classified as at high risk and had complete data over 3 years. At baseline, 2271 (39%) reported receiving AOM, 739 (13%) reported prior but not current use, and 2764 (48%) said they had never used AOM. Over 3 years, 85% of baseline non-users continued as non-users and 15% initiated AOM; among baseline users, 49% continued the same medication class, 29% stopped AOM, and 12% switched. Women who stopped AOM were less likely to self-report osteoporosis (HR 0.56, 95% CI 0.42–0.75) than women who continued AOM. Compared with non-users who did not begin treatment, women initiating AOM were more likely to report a diagnosis of osteoporosis (HR 11.3, 95% CI 8.2–15.5) or osteopenia (HR 4.1, 95% CI 2.9–5.7) and be very concerned about osteoporosis (HR 1.9, 95% CI 1.3–2.8).

**Conclusions:**

Less than 40% of women at high risk of fracture reported taking AOM. Women who stopped AOM were less likely to believe they have osteoporosis. Women who initiated treatment appeared motivated primarily by a diagnosis of osteoporosis or osteopenia and concern about the condition.

## Introduction

Anti-osteoporosis medications (AOMs) are efficacious in reducing risk of fractures in postmenopausal women [1]–[3]. Unfortunately, effective fracture prevention has been hampered by sub-optimal prescribing of medications to high-risk women [4]–[6] and low adherence among women who have started AOM [7]–[9]. Identifying factors associated with patterns of use of these medications has the potential to improve prescribing and adherence. Data from the large international Global Longitudinal Study of Osteoporosis in Women (GLOW) provide an opportunity to explore these associations.

We selected postmenopausal women at high risk of major fracture, as determined by the World Health Organization FRAX tool [10], [11], to describe patterns of AOM use during 3 years of observation and to identify characteristics associated with these patterns.

## Methods

### Ethics Statement

Each study site obtained ethics committee approval to conduct the study in the specific location.

### Study Design

GLOW is an observational cohort study conducted in physician practices in 17 sites in 10 countries (Australia, Belgium, Canada, France, Germany, Italy, Netherlands, Spain, UK, and USA). Details of the study design and methods have been described previously [12]. In brief, study sites were selected based on geographic distribution and the presence of lead investigators with expertise in osteoporosis and access to a research team capable of managing a large cohort of subjects. Investigators identified primary care practices in their region that were able to supply names and addresses of their patients electronically. The composition of groups varied by region and included health-system owned and independent practices and health maintenance organizations. Each practice provided a list of the names and addresses of women aged ≥55 years who had been attended by their physician in the past 24 months. All eligible women aged ≥65 years and a random sample of 50% of women <65 years of age were recruited from each practice. Patients who were unable to complete the study survey due to cognitive impairment, language barriers, institutionalization, or illness were not included.

Questionnaires were designed to be self-administered and covered domains including: patient characteristics and risk factors; perception of fracture risk and osteoporosis; medication use (current or ever taken); selected medical diagnoses; healthcare access and use; physical activity; and physical and emotional health status. Where possible, items from validated instruments were used, including the National Health and Nutrition Examination Survey (NHANES) [13], EuroQol (EQ-5D) [14], [15], and the Physical Function Component of the SF-36 [16]–[18].

All information was self-reported. For the baseline survey, subjects were asked to identify fractures they had experienced since the age of 45 years for any of 10 specified locations: clavicle, upper arm, wrist, spine, rib, hip, pelvis, upper leg, lower leg, and ankle. When more than one fracture was reported, each was counted to obtain the total fractures by site. Women’s self-reports of prior fracture were not validated from independent records.

FRAX scores were calculated for all women from responses on their baseline surveys. Women missing variables required to calculate a FRAX score were excluded from further analysis. Women were classified as “high risk” if their FRAX 10-year probability of major fracture was ≥20%.

AOMs were grouped as: oral bisphosphonates (alendronate, etidronate, ibandronate, risedronate); bisphosphonate infusion (pamidronate, zolendronic acid); and parathyroid hormone. All other classes of AOM included only one medication and were analyzed separately, and included calcitonin, raloxifene, strontium ranelate, and tibolone. Estrogen-containing medications that may have been prescribed as hormone-replacement therapy were not included.

AOM usage categories were formed for women who reported one class of AOM in each survey year (baseline, year 1, year 2, and year 3). Women who reported current AOM use at baseline were considered as having “Stopped AOM” at the first survey with no current use reported. Women who reported current AOM use at baseline and a different class in a subsequent survey were considered as having “Switched AOM” in the year of change. Women who both stopped and switched were counted only as having switched. Women who reported no current use of AOM at baseline but current use in a later year were designated as “Initiated AOM” in the first year of current use. Women who reported current use of the same class of AOM in each survey year were designated as “Continuous use, same class,” and women who reported no past use of AOM at baseline and no current use during any survey year were designated as “Never used AOM.” Women missing any survey or with incomplete AOM or outcome data were excluded from the analysis.

Separate models were fit for the outcomes of stopping, starting, and switching AOM use using multiple Cox regression. Risk factors whose status could change over survey years were modeled as time-varying covariates (TVCs). Because the relative timing of TVC and outcome was not known if they co-occurred in the same survey year, model log likelihoods were compared for models where TVC status in year t was determined solely by its status in year t (same survey year as outcome); solely by its status in year t–1 (prior survey); and by its status in year t–1 and year t (year t status yes, if yes in prior or same year as outcome). In most instances it is reasonable to suppose the TVC precedes the outcome if they co-occur; however, if the improvement in model likelihood under a different timing assumption had a p-value ≤0.05 (chi-square test), the improved model was reported.

All three models were fit separately using backwards stepwise selection, beginning with all variables identified as significant (p<0.20) in the univariate analyses. These are shown in Table S1. Country was also included in each model as a fixed effect. Variables that remained significant (p≤0.05) were retained in the final model. The c index for each model was computed using the Harrell macro for Cox regression [19]. This macro was modified to incorporate, where applicable, changing risk factor status over the survey years. All analyses were conducted in SAS versions 9.2 and 9.3 (SAS Institute, Cary, NC).

## Results

**Figure 1:**
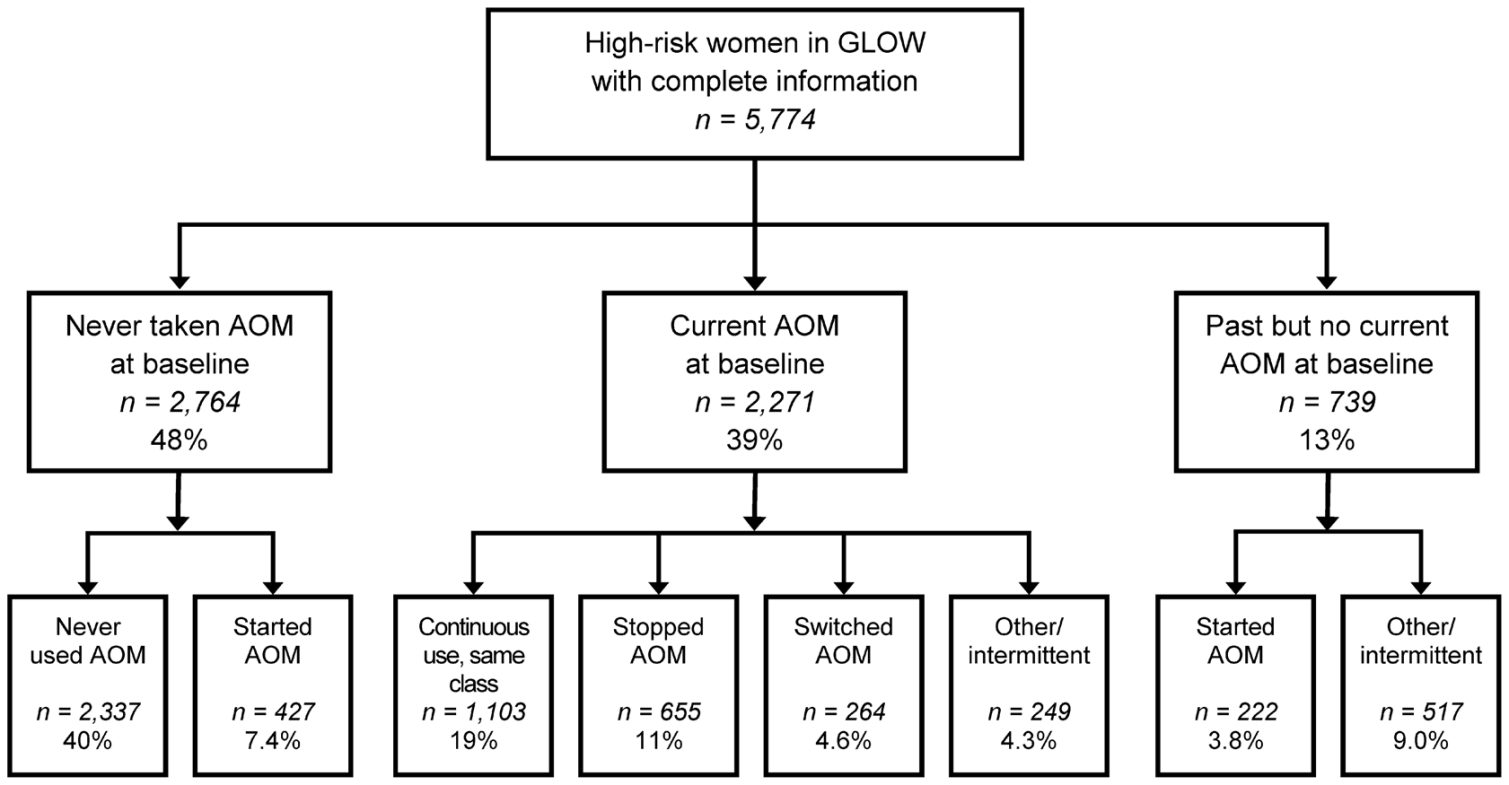
Patient flow chart.

Among the >100,000 women mailed an invitation to participate, the median participation rate across the 17 study sites was 62%. At study baseline, 40,228 women had recorded FRAX scores; 8532 (21%) had a 10-year probability ≥20% of sustaining a major fracture and were designated “high risk”. Of the high-risk women, 5774 (68%) had complete fracture and medication follow-up data through survey year 3 and comprise the manuscript data set. At baseline, 2764 (48%) women said they had never used AOM; 2271 (39%) women reported current use of an AOM; and 739 (13%) said they were not taking AOM now but had in the past (Figure 1).

Over the ensuing 3 years, 2337 (85%) of baseline non-users continued as non-users and 427 (15%) initiated use (Figure 1). Among baseline users, 1103 (49%) continued use of the same class of medication for the entire follow-up, 655 (29%) stopped and did not restart use, 264 (12%) switched class of medication, and 249 (11%) reported intermittent use. Of the women reporting past but not current use at baseline, 517 (70%) continued as non-users or intermittent users and 222 (30%) re-started AOM.

Table 1 displays the baseline risk characteristics for the lower-risk and high-risk women. High-risk women were on average older and had lower weight and height. Frequencies of FRAX risk factors were greater among high-risk women, with the exceptions of cigarette smoking and increased alcohol use.

**Table 1:** Baseline characteristics of the lower-risk and high-risk populations.

	Lower-risk women(n = 22,002)	High-risk women(n = 5774)
Age, years	64 (60–70)	76 (71–81)
Weight, kg	68 (60–79)	63 (56–71)
Height, cm	163 (157–166)	160 (155–165)
Previous fracture^a^	2430 (11)	3614 (63)
Parental hip fracture	2783 (13)	2279 (39)
Current smoking	1668 (7.6)	394 (6.8)
Glucocorticoid use	339 (1.5)	407 (7.1)
Rheumatoid arthritis	126 (0.6)	112 (1.9)
Alcohol consumption >20 units per week	106 (0.5)	32 (0.6)
Secondary osteoporosis^b^	3686 (17)	1620 (28)

Data are median (interquartile range) or count (percentage).

^a^Fracture since age 45 of clavicle or collar bone, upper arm, wrist, spine, rib, hip, pelvis, ankle, upper leg, or lower leg.

^b^Type 1 diabetes; menopause before age 45 years; diagnosis of ulcerative colitis or celiac disease; or current use of anastrozole, letrozole, or exemestane.

Table S1 summarizes results of univariate analyses of high-risk women who altered their AOM use during the course of the study by stopping AOMs, switching class of AOM, or starting medication. Compared with those who used AOM continuously, women who stopped their medication were less likely to be ≥75 years of age, to have received a diagnosis of osteoporosis, to report fair or poor health, to be very concerned about osteoporosis, or rate their fracture risk as higher than that of their peers.

Also compared with those who used AOM continuously, women who switched their medication to a different class had lower body mass indexes, were more likely to have fallen, and reported more multiple fractures both at baseline and in the preceding 12 months. HRs were >1 for most fracture sites, again at baseline and in the preceding 12 months (Table S1). These women had a higher self-reported rate of secondary osteoporosis, a greater frequency of co-occurring conditions, and more often said they had been diagnosed with osteoporosis. They were also more likely to say that their health was fair or poor, expressed greater concern about osteoporosis, and rated their perceived risk of fracture as increased relative to that of their peers.

Compared with those who never used AOM, women who initiated treatment had lower body mass indexes, were more likely to report a fall in the preceding 12 months, and reported a greater frequency of baseline and incident fractures (Table S1). They were more often current glucocorticoid users and more often reported diagnoses of osteoporosis and osteopenia. General health was more likely to be rated fair or poor, physical function was somewhat lower, and greater concern about osteoporosis and fracture risk was noted.

**Figure 2:**
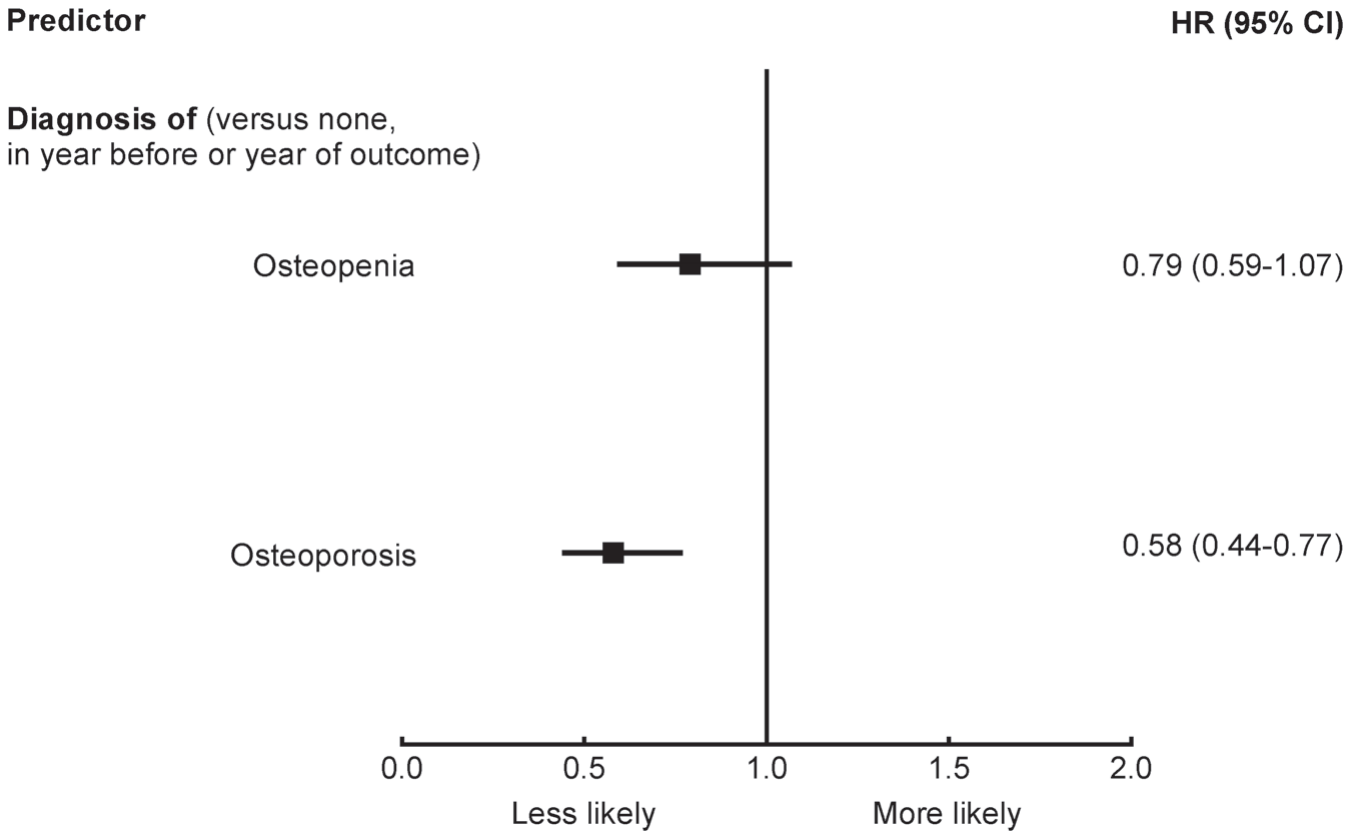
Multivariable HRs predicting stopping AOM (c = 0.58). AOM, anti-osteoporosis medication; CI, confidence interval; HR, hazard ratio.

**Figure 3:**
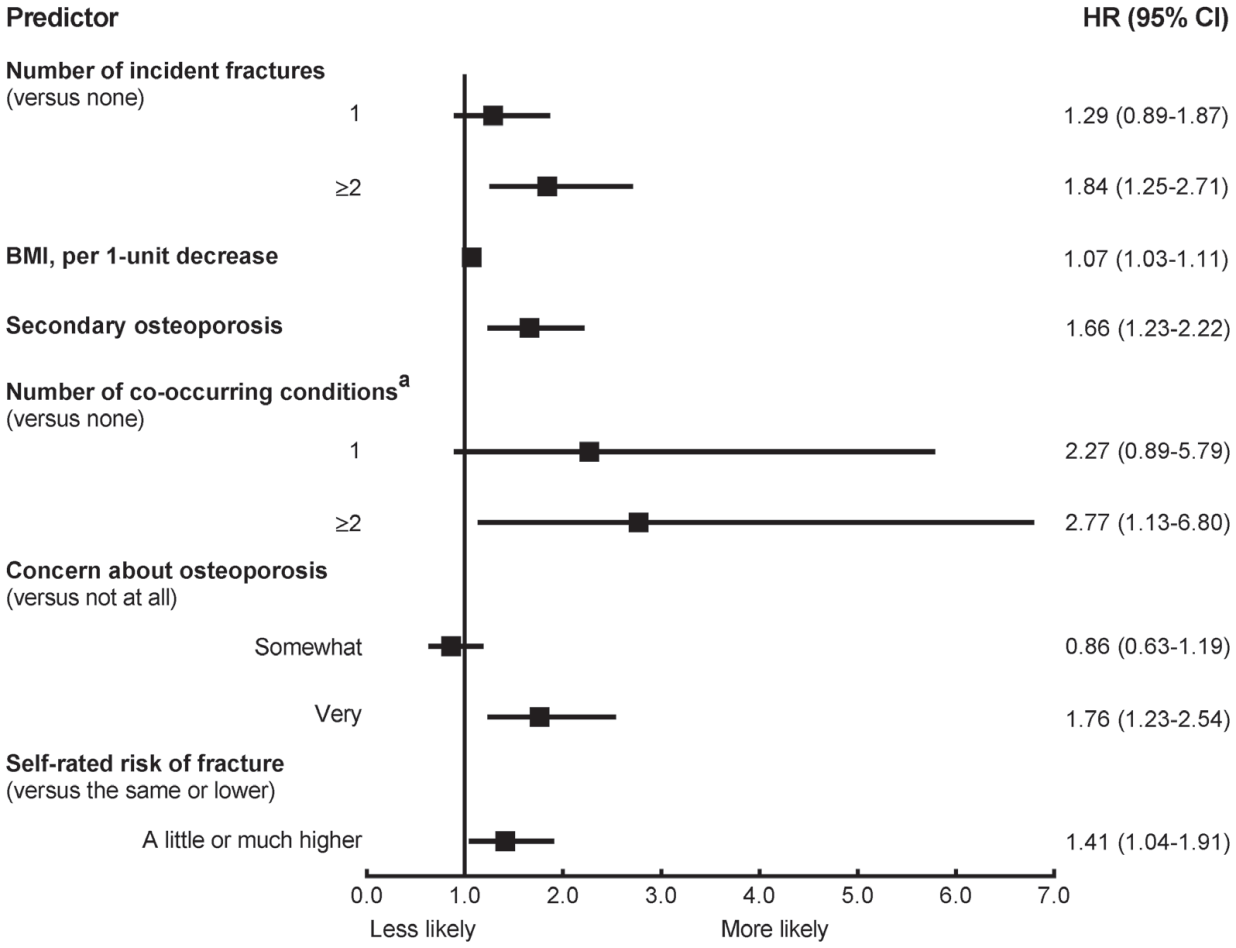
Multivariable HRs predicting switching AOM (c = 0.70). AOM, anti-osteoporosis medication; BMI, body mass index; CI, confidence interval; HR, hazard ratio.^ a^Asthma, chronic bronchitis or emphysema, osteoarthritis or degenerative joint disease, rheumatoid arthritis, stroke, ulcerative colitis or Crohn’s, celiac disease, Parkinson’s disease, multiple sclerosis, cancer, type 1 diabetes, hypertension, heart disease, high cholesterol concentration.

**Figure 4:**
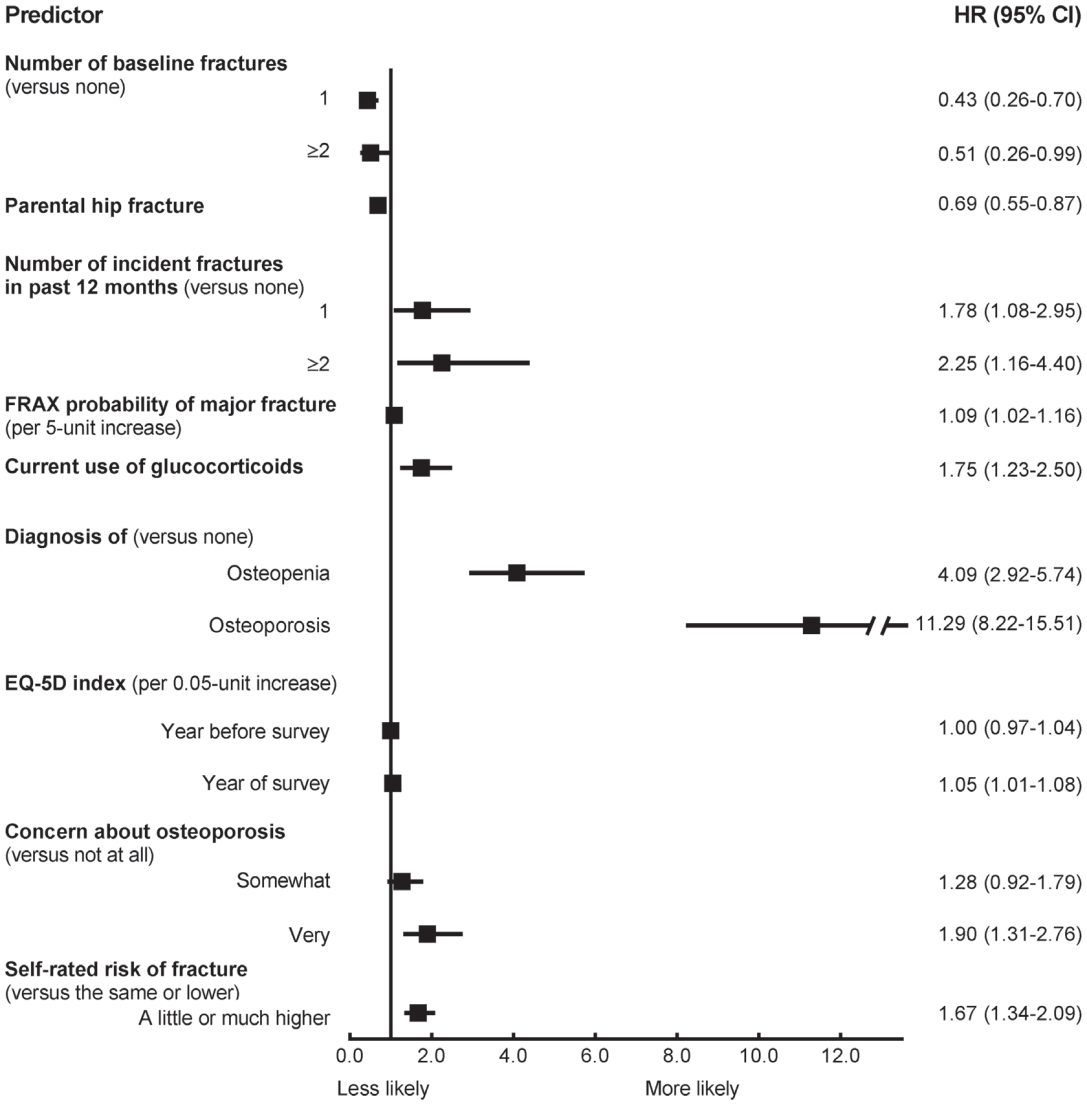
Multivariable HRs predicting starting AOM (c = 0.77). AOM, anti-osteoporosis medication; CI, confidence interval; HR, hazard ratio.

Results of multivariable modeling to identify factors independently associated with patterns of use are shown in Figures 2–4. The only factor independently associated with an increased likelihood of stopping medication was the absence of the diagnosis of osteoporosis (Figure 2) (i.e. women reporting a diagnosis of osteoporosis were less likely to discontinue AOM). The c-index of 0.58 for the model demonstrated little discrimination between those who stopped and those who continued AOM [20].

Characteristics associated with switching AOM included multiple incident fractures, decreasing body mass index, a diagnosis compatible with secondary osteoporosis, and multiple co-occurring conditions (Figure 3). Heightened concern about osteoporosis and risk of fracture were also predictors for switching. The c-index for the model was 0.70.

Most strongly associated with initiating AOM treatment was having a diagnosis of osteoporosis or osteopenia (Figure 4). Incident fractures in the past 12 months, increasing EQ-5D score, and current use of glucocorticoids were associated with starting treatment, as were increased levels of concern about osteoporosis or risk of fracture (Figure 4). Reports of prior fractures at baseline and history of parental hip fracture were negatively associated with starting medication. The c-index for the model demonstrated moderate discrimination (0.77).

## Discussion

In this large, international cohort of high-risk postmenopausal women, we looked for characteristics that could help explain patterns of medication use. Among nearly 6000 women, only 39% were taking an AOM at baseline and 48% had no current or past exposure to the medications. Over the 3 years of observation, 49% of baseline AOM users continued use of their baseline class of AOM and 85% of non-users continued as non-users. Among the baseline AOM users, 29% stopped their medication and 12% switched class of medication. Nineteen percent of baseline non-users initiated treatment.

Multivariable models identified several established risk factors, including low body mass index, prior fractures, and use of glucocorticoid that contributed to the identification of women who initiated and who switched medications. Women who reported a history of a previous fracture or that a parent had suffered a hip fracture on the baseline survey were less likely to start AOM. This apparent paradox may reflect a continuing decision of a subgroup of women who have already considered these risk factors and elected to forego AOM treatment. Only the absence of a diagnosis of osteoporosis was significantly associated with stopping AOM, and the low c-index (0.58) indicated that modeling was unable to produce factors that identified women who were likely to be non-persistent. FRAX variables were generally less robust predictors of medication behavior than factors such as a diagnosis of osteoporosis, concern about osteoporosis, and self-perceived fracture risk. Two FRAX variables, cigarette smoking and heavy alcohol use, showed similar frequencies among high risk and lower risk women (Table 1) and also failed to predict medication use. It appears that women with clinical risk factors may not recognize the underlying fracture risk they are carrying and/or that physicians may not actively screen women for fracture risk. The findings also suggest that patterns of use may be driven less by reactions to specific risk factors than by the labeling of the condition and a woman’s sense of susceptibility to osteoporosis and fracture. Indeed, some physicians may be reluctant to offer AOM to women who do not meet bone mineral density criteria for a diagnosis of osteoporosis given controversy over the efficacy of treating patients with bone density results above this level [21]–[24].

Our observations are consistent with other research. There is repeated evidence that most women with increased risk of fracture go untreated [25]–[27]. In a previous report from GLOW, Greenspan et al. [25] found that just 17% of treatment-naive women with a new fracture had begun an AOM in the first year of follow-up. Furthermore, Bessette et al. [26] found that only 26% of women who sustained fragility fractures had been treated within 6–8 months of the event. Lastly, Ryder et al. [27] reported that only 13% of older, community-dwelling women with low bone density indications for anti-fracture therapy were taking antiresorptive medication.

In previous studies, the patient characteristics most strongly associated with treatment initiation include low bone density and/or the diagnosis of osteoporosis (both documented and self-reported) [25]–[30]. In the current study, self-report of osteoporosis was the strongest predictor for initiating treatment, but we did not examine bone density. Older age, [26], [29], low body mass index [29], specific fracture sites (hip, spine, femur, pelvis) [25], [26], and use of calcium and/or vitamin D supplements [25], [26], have also predicted treatment in some studies. Similarly to the current study, health beliefs such as susceptibility to osteoporosis and efficacy of AOM treatment have been found by others [30], [31].

Declining persistence in taking osteoporosis medications over time has also been repeatedly demonstrated [7], [9]. In a systematic review that included 14 databases, Cramer et al. [7] found that persistence with bisphosphonate treatment for osteoporosis at 1 year ranged from 18% to 78%. Kothawala et al. [9] reported that, typically, only 50% of women prescribed an AOM are taking the medicine 12 months later.

Factors that influence persistence with therapy are similar to those related to initiation [7], [8], [32]–[34]. Women with a diagnosis of osteoporosis or low bone mineral density [32]–[34] and those with a previous fracture [8], [33] are most likely to persist with treatment. Younger women tend to be more persistent, [8] as are women with belief in the efficacy of medication [34]. Silverman et al. [35] noted that women who do not adhere to medications “may not believe that they have osteoporosis or that they are not at much risk of fracture,” an observation consistent with our findings.

Much research has utilized data from administrative databases to explore the effect of dosing regimens (daily versus weekly) on adherence [7], [8]. These sources are limited to measuring variables such as prescription refills and medication possession ratios and cannot explore patient attitudes and concerns that may influence adherence. We did not collect dosing information from our subjects, but we were able to assess women’s concerns about osteoporosis and perceived risks of fracture, which had a strong influence on medication use.

### Limitations and Strengths

Firstly, our data related to medication use are self-reported and have not been confirmed by pharmacy records. However, a recent report showed reasonable agreement when patient self-report of use of osteoporosis drugs was compared with pharmacy data [36], and a review by Garber et al. [37] found that self-reports of medication adherence from questionnaires had moderate-to-high concordance with electronic measures. Secondly, diagnoses of osteoporosis and osteopenia were not verified. There is likely both some over- and some under-reporting of both conditions. It is, however, less the “accurate diagnosis of osteoporosis” than “patient perception of the disease” that appears to drive behavior.

Patient reports of medication behaviors reflect the combined decisions of themselves and their physicians. A woman can only be on treatment if her doctor prescribes a medication *and* she elects to take it. Conversely, she may decide to discontinue AOM herself or with her doctor’s advice. Therefore, having only subject reports, we were unable to identify the sources of any decisions.

Each survey represents a cross-sectional assessment, hence, determining antecedent–consequent relationships is not possible. For example, heightened concern about osteoporosis may either precede and precipitate initiation of treatment or may be a consequence of the decision to begin AOM. However, in most cases, a woman’s attributes, attitudes, and actions are clustered in a way that such distinctions are not critical to understanding behavior.

Our high-risk designation for women was based on estimates derived from the FRAX tool which, although based on well-established risk factors for fracture, was not published until 2008. As our baseline collection of data took place in 2007, clinicians would not have had FRAX algorithms available for assessing their patients. However, clinical risk factors associated with fracture were recognized and acknowledged well before the publication and dissemination of FRAX [38].

The strengths of this study include the large sample size and the uniform method of collecting data across study sites. Data were gathered from patients of primary care physicians and there were few exclusion criteria. Physicians did not select specific patients for this study; they merely provided lists of active patients so the overall group to whom the questionnaires were sent initially should be representative of the practices.

## Conclusions

Many women who are at high risk of fracture are not taking medication that could reduce their risk. Those who do initiate treatment are more likely to self-report a diagnosis of osteoporosis or osteopenia, and appear to be motivated primarily by concern about osteoporosis and risk of fracture. Women who stop their medication are more likely to believe they do not have osteoporosis.

## Supporting Information

Table S1 **Univariate HRs for stopping, switching, or starting AOM among high-risk women.**
(DOCX)
