# Supplementary material for: Patterns of Anti-Osteoporosis Medication Use among Women at High Risk of Fracture: Findings from the Global Longitudinal Study of Osteoporosis in Women (GLOW)
Source: PLoS One. 2013 Dec 20;8(12):e82840. doi: 10.1371/journal.pone.0082840 (PMC3869728; doi:10.1371/journal.pone.0082840)
Supplement: Table S1 — Univariate HRs for stopping, switching, or starting AOM among high-risk women. (DOCX) [file pone.0082840.s001.docx]

Table S1. Univariate HRs for stopping, switching, or starting AOM among high-risk women.

|  | Stopped (vs continuous)  (n=655) | Switched (vs continuous) (n=264) | Started (vs never used) (n=649) | |  |
| --- | --- | --- | --- | --- | --- |
| Risk factors for fracture |  |  |  | |  |
| Baseline age, per 1-year increase | 0.99 (0.98–1.00) | 0.98 (0.97–1.00)* | 1.01 (1.00–1.02) | |  |
| Baseline age ≥75 years (vs <75 years) | 0.84 (0.72–0.98)* | 1.01 (0.79–1.30) | 1.13 (0.96–1.32)* | |  |
| BMI, per 1-unit decrease | 0.99 (0.97–1.00)* | 1.05 (1.02–1.09)* | 1.03 (1.01–1.05)* | |  |
| BMI <22 kg/m^2^ (vs ≥22 kg/m^2^) | 1.01 (0.85–1.19) | 1.27 (0.98–1.65) | 1.19 (0.99–1.43)* | |  |
| Baseline FRAX score, per 5-unit increase | 1.01 (0.97–1.05) | 1.08 (1.02–1.14)* | 1.10 (1.05–1.15)* | |  |
| Falls in past 12 months (vs 0) |  | *^,a^ | *^,a,b^ | |  |
| 1 | 0.91 (0.75–1.11) | 1.41 (1.06–1.88) | 1.17 (1.00–1.37) | |  |
| ≥2 | 0.99 (0.79–1.24) | 1.49 (1.08–2.06) | 1.34 (1.12–1.61) | |  |
| Number of fractures at baseline (vs 0) |  | * | * | |  |
| 1 | 1.03 (0.86–1.22) | 1.25 (0.92–1.70) | 1.18 (1.00–1.41) | |  |
| ≥2 | 0.95 (0.76–1.18) | 2.04 (1.47–2.83) | 2.33 (1.88–2.88) | |  |
| Baseline fracture |  |  |  | |  |
| Collar bone or clavicle | 0.84 (0.53–1.34) | 1.25 (0.68–2.29) | 1.00 (0.66–1.51) | |  |
| Upper arm | 0.85 (0.65–1.11) | 1.07 (0.73–1.56) | 1.50 (1.13–1.98) | |  |
| Wrist | 1.03 (0.87–1.22) | 1.24 (0.95–1.61) | 1.32 (1.11–1.57) | |  |
| Spine | 0.91 (0.68–1.21) | 2.50 (1.86–3.35) | 3.48 (2.65–4.57) | |  |
| Rib | 0.99 (0.79–1.25) | 1.42 (1.12–2.06) | 1.29 (1.01–1.65) | |  |
| Hip | 1.01 (0.74–1.37) | 1.86 (1.29–2.67) | 1.94 (1.41–2.67) | |  |
| Pelvis | 1.01 (0.69–1.48) | 1.55 (0.96–2.49) | 2.11 (1.38–3.22) | |  |
| Ankle | 0.98 (0.79–1.22) | 0.98 (0.70–1.37) | 0.87 (0.69–1.08) | |  |
| Upper leg | 0.97 (0.61–1.55) | 1.37 (0.75–2.50) | 2.44 (1.59–3.73) | |  |
| Lower leg | 1.06 (0.77–1.46) | 1.53 (1.02–2.32) | 1.28 (0.95–1.72) | |  |
| Number of incident fractures (vs 0) |  | * | * | |  |
| 1 | 1.07 (0.90–1.28) | 1.23 (0.90–1.70) | 1.31 (1.09–1.57) | |  |
| ≥2 | 0.92 (0.74–1.15) | 2.11 (1.53–2.93) | 2.58 (2.09–3.17) | |  |
| Incident fracture in past 12 months |  |  |  | |  |
| Collar bone or clavicle | 1.04 (0.26–4.17) | 2.19 (0.55–0.88) | 1.68 (0.42–6.73) | |  |
| Upper arm | 0.92 (0.23–3.66) | 4.65 (1.91–11.28)* | 3.04 (1.67–5.51)* | |  |
| Wrist | 0.92 (0.44–1.93) | 1.62 (1.12–2.35)*^,a,b^ | 2.52 (1.53–4.14)* | |  |
| Spine | 1.04 (0.49–2.18) | 2.61 (1.80–3.79)*^,a,b^ | 4.02 (2.97–5.44)*^,a,b^ | |  |
| Rib | 0.91 (0.41–2.04) | 2.15 (0.96–4.83)* | 2.15 (1.07–4.31)* | |  |
| Hip | 1.69 (0.70–4.08) | 2.75 (1.76–4.28)*^,a,b^ | 3.59 (1.92–6.72)* | |  |
| Pelvis | 1.36 (0.56–3.27) | 1.35 (0.34–5.43) | 5.98 (2.48–14.41)* | |  |
| Ankle | ^c^ | 1.67 (0.54–5.21) | 3.87 (2.13–7.03)* | |  |
| Upper leg | 1.93 (0.86–4.30)* | 3.19 (1.02–9.96)* | 2.06 (1.19–3.58)*^,a,b^ | |  |
| Lower leg | 0.84 (0.12–5.95) | ^c^ | 0.67 (0.10–4.76) | |  |
| Baseline current cigarette use | 1.22 (0.90–1.65) | 1.00 (0.59–1.72) | 1.02 (0.76–1.37) | |  |
| Baseline history of parental hip fracture | 1.10 (0.94–1.28) | 0.87 (0.68–1.13) | 0.76 (0.65–0.89)* | |  |
| Current use of glucocorticoid | 0.89 (0.64–1.24) | 1.39 (0.93–2.08)* | 2.59 (1.97–3.42)* | |  |
| Secondary osteoporosis^d^ | 1.08 (0.90–1.29) | 1.66 (1.28–2.15)* | 1.04 (0.88–1.25) | |  |
| Baseline alcohol use ≥20 units per week | 2.66 (0.66–10.64)* | 3.10 (0.44–22.12) | 0.37 (0.09–1.49)* | |  |
| No. of co-occurring conditions^e^ (vs 0) | * | * | * | |  |
| 1 | 0.72 (0.52–1.00) | 2.31 (1.05–5.10) | 1.08 (0.77–1.52) | |  |
| ≥2 | 0.97 (0.73–1.29) | 2.97 (1.40–6.30) | 1.37 (1.01–1.85) | |  |
| Diagnosis of (vs 0) | * | * | * | |  |
| Osteoporosis | 0.60 (0.45–0.79) | 3.11 (1.28–7.54) | 15.24 (11.95–19.44) | |  |
| Osteopenia | 0.90 (0.67–1.23) | 1.64 (0.64–4.22) | 4.17 (3.15–5.53) | |  |
| General health fair or poor | 0.79 (0.64–0.97)* | 1.33 (1.01–1.76)* | 1.85 (1.55–2.20)* | |  |
| SF-36 physical function, per 10-unit increase | 1.02 (0.99–1.05)* | 0.94 (0.90–0.98)* | 0.93 (0.91–0.96)* | |  |
| SF-36 vitality, per 10-unit increase | 1.03 (0.99–1.07)* | 0.96 (0.90–1.02) | 0.93 (0.89–0.96)* | |  |
| EQ-5D index, per.05-unit increase | 1.01 (0.98–1.03) | 0.98 (0.95–1.01)* | 0.97 (0.94–1.00)^a^  0.97 (0.95–1.00)^b^  *^,a,b^ | |  |
| Concern about osteoporosis (vs not at all) | * | *^,a,b^ | * | |  |
| Very | 0.70 (0.54–0.92) | 1.83 (1.35–2.48) | 5.70 (4.39–7.40) | |  |
| Somewhat | 0.83 (0.65–1.06) | 0.84 (0.63–1.11) | 2.06 (1.60–2.65) | |  |
| Self-rated risk of fracture compared to other women of the same age (vs the same or lower) |  |  | |  | |
| A little or much higher | 0.84 (0.71–0.98)* | 1.88 (1.47–2.40)* | 3.31 (2.82–3.89)* | |  |

Data are HR (95% CI).

AOM, anti-osteoporosis medication; BMI, body mass index; CI, confidence interval; HR, hazard ratio.

* p<0.20.

^a^ Response in survey year before outcome used.

^b^ Response in survey year of outcome used.

^c^ Fracture numbers too small to calculate

^d^ Type 1 diabetes; menopause before age 45 years; diagnosis of ulcerative colitis or celiac disease; or current use of anastrozole, exemestane, or letrozole.

^e^ Asthma, chronic bronchitis or emphysema, osteoarthritis or degenerative joint disease, rheumatoid arthritis, stroke, ulcerative colitis or Crohn’s disease, celiac disease, Parkinson’s disease, multiple sclerosis, cancer, type 1 diabetes, hypertension, heart disease, high cholesterol.
